# Stress experiences in neighborhood and social environments (SENSE): a pilot study to integrate the quantified self with citizen science to improve the built environment and health

**DOI:** 10.1186/s12942-018-0140-1

**Published:** 2018-06-05

**Authors:** Benjamin W. Chrisinger, Abby C. King

**Affiliations:** 1 Stanford Prevention Research Center, Department of Medicine, School of Medicine, Stanford University, 1070 Arastradero Road, Suite 100, Palo Alto, CA 94304 USA; 2 Department of Health Research and Policy, School of Medicine, Stanford University, Palo Alto, USA

**Keywords:** Citizen science, Quantified self, Chronic stress, Allostatic load, Electrodermal activity, Built environment, Sensors, Leaflet

## Abstract

**Background:**

Identifying elements of one’s environment—observable and unobservable—that contribute to chronic stress including the perception of comfort and discomfort associated with different settings, presents many methodological and analytical challenges. However, it also presents an opportunity to engage the public in collecting and analyzing their own geospatial and biometric data to increase community member understanding of their local environments and activate potential environmental improvements. In this first-generation project, we developed a methodology to integrate geospatial technology with biometric sensing within a previously developed, evidence-based “citizen science” protocol, called “Our Voice.” Participants used a smartphone/tablet-based application, called the Discovery Tool (DT), to collect photos and audio narratives about elements of the built environment that contributed to or detracted from their well-being. A wrist-worn sensor (Empatica E4) was used to collect time-stamped data, including 3-axis accelerometry, skin temperature, blood volume pressure, heart rate, heartbeat inter-beat interval, and electrodermal activity (EDA). Open-source R packages were employed to automatically organize, clean, geocode, and visualize the biometric data.

**Results:**

In total, 14 adults (8 women, 6 men) were successfully recruited to participate in the investigation. Participants recorded 174 images and 124 audio files with the DT. Among captured images with a participant-determined positive or negative rating (n = 131), over half were positive (58.8%, n = 77). Within-participant positive/negative rating ratios were similar, with most participants rating 53.0% of their images as positive (SD 21.4%). Significant spatial clusters of positive and negative photos were identified using the Getis-Ord Gi* local statistic, and significant associations between participant EDA and distance to DT photos, and street and land use characteristics were also observed with linear mixed models. Interactive data maps allowed participants to (1) reflect on data collected during the neighborhood walk, (2) see how EDA levels changed over the course of the walk in relation to objective neighborhood features (using basemap and DT app photos), and (3) compare their data to other participants along the same route.

**Conclusions:**

Participants identified a variety of social and environmental features that contributed to or detracted from their well-being. This initial investigation sets the stage for further research combining qualitative and quantitative data capture and interpretation to identify objective and perceived elements of the built environment influence our embodied experience in different settings. It provides a systematic process for simultaneously collecting multiple kinds of data, and lays a foundation for future statistical and spatial analyses in addition to more in-depth interpretation of how these responses vary within and between individuals.

**Electronic supplementary material:**

The online version of this article (10.1186/s12942-018-0140-1) contains supplementary material, which is available to authorized users.

## Background

Living and working in chronically stressful environments are thought to contribute to a wide range of adverse health outcomes. While the body’s stress response systems (e.g., “fight-or-flight”) may be helpful in adapting to acute environmental stimuli, major life events, or trauma, the continual triggering of these mechanisms may diminish an individual’s biological capacity to respond to stressors [1]. With the impairment of this biological system, chronic stress can increase an individual’s risk of experiencing adverse health outcomes, including chronic diseases such as obesity and type II diabetes [1–3]. Emerging research is also beginning to help us conceptualize and document the causal pathways, including stress, related to built environments that may explain the well-documented burden of chronic disease in disadvantaged communities [4–6].

Many studies exploring connections between stress and health have used levels of blood or salivary cortisol, a steroid hormone produced as part of the body’s stress response. Most cortisol research attempts to describe cumulative stress effects, though additional attention to the different types of stressors—events, structural circumstances, or daily routines—is needed to understand possible pathways to poor health [7]. For example, prior studies often have found limited or conflicting evidence about the nature of the relationship between cortisol and socioeconomic status [7]. McEwen and colleagues proposed a broad framework for measuring chronic stress that included a set of ten biometric markers, including cortisol, a steroid hormone produced as part of the body’s stress response. Called “allostatic load” (AL), the measure was intended as a proxy measure for overall “wear and tear” on the body’s stress response systems [1]. Other researchers have found relationships between AL and adverse mental and behavioral health outcomes, including cognition [2, 8].

The concept of allostatic load has been applied to chronic environmental stressors, especially those related to workplace or neighborhood environments [8, 9]. Theall et al. [8] found that a significant amount of AL variance among adolescents in the National Health and Nutrition Examination Survey was attributable to neighborhood-level factors, such as poverty, crime, and density of alcohol retailers, even after controlling for household-level characteristics. While biomarkers such as cortisol or allostatic load allow for a consideration of the cumulative effects of stress, they are not as well suited to understanding the relative influence of different stressors, or how individual and environmental characteristics intersect to yield different stress outcomes.

In response to this challenge, Roe and colleagues have spearheaded a new generation of interdisciplinary research that measures direct biological responses to different types of built environments. Using a head-worn device, the Emotiv EPOC, Roe et al. [10] explored changes in brain activity (measured via electroencephalogram, or EEG) as laboratory participants were exposed to images of urban and natural landscape scenes. These findings informed later EEG studies with free-moving participants walking in different types of environments. For instance, Aspinall et al. [11] found EEG changes as participants moved into and out of urban green spaces and other environments.

Complicating the identification of environmental contributors to chronic stress is the role of individual perception [12]. For instance, environmental health researchers have found that individuals living in objectively noisy neighborhoods do not, in general, exhibit the same elevated stress responses to noise as those that do not. That is, individuals may become habituated to or learn to alter their perceptions of such chronically stressful environments [13], although resident selection factors may also be at work, at least to some extent (e.g., individuals who are particularly sensitive to high noise levels may move out of or avoid such neighborhoods) [14–19]. Based on such research, studies aimed at investigating physiological responses to stress in differing environments are challenged to integrate perception-based measures within their built environment assessments. For example, Aspinall et al. [11] also asked participants to rate scenes across several subjective criteria (e.g., pleasure/displeasure, calm/excitement), adding a dimension of personal preference and underlying attitudes toward certain types of built environments.

One relatively untested arena that may help us understand both individual characteristics and perceptions that matter for chronic stress is the *quantified self.* This sensor-oriented movement encompasses a range of self-monitoring technologies, especially those that can be integrated into smartphones as mobile applications [20]. Users typically collect and review biometric (e.g., weight, heart rate) and/or behavioral (e.g., diet, sleep, exercise) data out of curiosity or an interest in self-improvement [20–22]. While quantified self applications may focus on individual or group-level changes in behavior through biofeedback or goal-setting, there is great potential to develop relevant insights on population-level outcomes given sufficiently large datasets [23, 24]. For instance, a recent smartphone-derived “big data” study found city-level correlations between objective walkability metrics and device-based walking outcomes measured from over 700,000 smartphone users across 111 countries [24].

Aside from proprietary wearable datasets, some university-sponsored projects aim to crowd-source place-based biometric data. One example is the Personal Activity Location Monitoring System (PALMS), which provides validated tools and methodologies for collecting geo-located biometric data to track behavior across space and time, especially individual and group-level dynamics of physical activity [25–27]. Another suite of projects employ “People as Sensors” methodologies that crowd-source a variety of objective and perceived data, including biometrics, in order to deliver relevant feedback to urban designers and planners [28–30]. For example, Zeile and colleagues used a biosensor-oriented approach to track how and why stress responses changed over space and time among a cohort of bicyclists in Cambridge, Massachusetts. Importantly, they found participants’ mapped stress data corresponded to individual experiences, as measured with video recordings [30].

Our pilot study builds upon earlier biometric built environment assessments by integrating dimensions of the quantified self movement. First, we utilized a biometric measure of stress involving electrodermal activity (EDA), which has been shown to be an effective means of differentiating between different kinds of stress environments and situations (e.g., driving in traffic vs. highway driving), and can be collected with relatively low impact on participant experience compared to head- or chest-worn devices [31]. Second, we embedded our biometric data collection within an existing successful “citizen science”-based community activation model, called “Our Voice”, which includes a mobile application, referred to as the “Discovery Tool,” that allows community members to collect objective and perceived neighborhood data [32, 33], providing a systematic and technology-assisted enhancement to existing community-based qualitative research methods, such as Photovoice [34, 35]. By creating a simple and reliable method of collecting geolocated stress data while participants use the Discovery Tool in the field, we aim to amplify the known strengths of this type of citizen science model. Finally, we introduce open-source methods for visualizing and sharing perceived and objective participant data with participants for their inspection and interpretation, with some possible applications for future testing with additional spatial data.

The primary goals of this pilot study were to: (1) test the feasibility of including biometric data collection via wrist-worn sensors as part of the objective and perceived built environment data collection capabilities of the Discovery Tool mobile app being employed to obtain resident-collected information on local built environments; (2) test the acceptability of different biometric data visualization styles; and (3) explore possible options for testing relationships between documented elements of the built environment and biometric changes/outcomes.

## Methods

### Participant recruitment

To be eligible for the study, prospective participants had to be healthy adult volunteers living in or around San Francisco, California, and able to complete a relatively easy 20- to 25-min walk. Participants were recruited as a convenience sample in San Francisco through the networks of our San Francisco Bay Area community partner, an urban planning and design nonprofit called Place Lab, and through members of the research team. Walk appointments were scheduled for one of two days in July and September 2017.

### Biometric sensor

A wrist-worn biometric sensor, the Empatica E4, was selected for this study because it provides a commercially available, easy-to-use means of continuously collecting time-stamped biometric data, including stress response and other possible measures that could be used in subsequent feature identification algorithms. Data collected by the E4 include 3-axis accelerometry (which measures gravitational force on three spatial dimensions, allowing for a three-dimensional understanding of participant movement), skin temperature, blood volume pressure, heart rate, heartbeat inter-beat interval, and electrodermal activity (EDA). Once participants signed the study consent form, which was approved by the Stanford University Institutional Review Board, they were asked to put on and activate the sensor, which they wore during a 10-min pre-walk period. The purpose of this approximately 10-min pre-walk data collection was two-fold: (1) to allow for the sensor to make appropriate contact with the skin surface, and (2) to collect baseline electrodermal activity data for subsequent data analysis.

### Mobile built environment audit tool application

This investigation was intended to determine the initial feasibility and utility of adding biometric sensor data to the built environment data collected with the DT app, which is typically embedded within a broader citizen science community engagement research method called *Our Voice.* This method has been used successfully to study built and social environments in a variety of settings [32], and features the simple DT mobile application to capture photos, audio narratives, and participant-assigned valences for the specific built environment elements being captured (“Is this [built environment element] good or bad for the community?”). The DT app also captures the geocoordinates of the user’s walking route and a short demographic survey upon completion of a walk. It additionally collects latitude and longitude coordinates every second while participants use it, and all photos taken with the app has geo-coordinates embedded in their metadata. A web portal for viewing DT participant data allows the research team to download summary data for each walk, including the walk route and locations of all photos and audio recordings. The full *Our Voice* process (not included in this pilot study) involves collection of geo-tagged photos and audio narratives with the DT mobile app, followed by facilitated community meetings to identify shared themes and build community consensus, in partnership with identified stakeholders, around how to address environmental issues negatively impacting resident health and well-being and [32, 33, 36–39].

Depending on their preference, participants downloaded the DT from the Apple App Store or Google Play Store [40, 41], or used electronic tablets (Samsung Galaxy Tab E Lite 7”) that were made available to them and already contained the required DT app [42]. Participants were verbally instructed on how to use the DT app, and prompted to take photos and describe aspects of this neighborhood environment that they felt influenced their well-being or the functioning of these public spaces.

### Neighborhood walks

Based on our community partner’s interest in existing and future public space projects in a specific neighborhood of San Francisco, California, an approximately 20-min walking route (0.9 miles) was predetermined to take participants through a variety of different environments, including a small public green space, back alleys, and busy commercial streets. Participants were instructed to document anything along the route that they believed influenced their well-being or the functioning of these public spaces. A researcher accompanied groups of up to four participants at a time to direct them along the route and help troubleshoot any difficulties with the app or wearable sensor. Participants were also encouraged not to talk to one another during the neighborhood walks. Following the neighborhood walk, participants completed a short demographic survey embedded within the DT app, and returned the biometric sensor and tablet (if borrowed) to the investigators. Five groups participated over two separate days in August and October 2017, and group sizes ranged from one to four participants, depending on participant availability.

Application stability issues related to the large quantity of photos and audio recordings taken by some participants caused the DT app images from several walks (n = 6) to be lost, though for two walks where audio files were recovered, the research team was able re-create the image in Google Street View by using the approximate location and subject being described in the audio file. For one of the participants without photo/audio data, the research team was also unable to recover biometric data. Biometric data were successfully downloaded and processed for the remaining participants (n = 13).

### Data processing

Each participant’s EDA data were normalized by subtracting the minimum value and dividing by the range from their baseline data values, consistent with prior research using EDA data from the Empatica E4 biosensor [31]. To assist with comparisons between participants, each participant’s normalized EDA data were also centered (subtracting the mean) and scaled (dividing by the standard deviation of the centered data). To help identify sudden changes, or “peaks,” in EDA, a proprietary algorithm from the company was applied to help remove erroneous readings, or “noise,” possibly caused by sudden motions or other non-EDA-related issues with the sensor. Skin temperature, 3-axis accelerometry, and EDA data files were provided as algorithm inputs; outputs included time-stamped peaks in EDA with characteristics such as peak amplitude and wavelengths.

Simple text processing was performed on participant audio narrative transcriptions using functions from the *tm* (“text mining”) package in R. To prepare the text for review, all letters were shifted to lowercase, very common words were removed, and a word frequency table was generated [43, 44]. This table was further grouped by nouns and adjectives, and words with a frequency greater than five were included in a visualization to compare the most prevalent terms across all participants.

### Data visualization

**Fig. 1:**
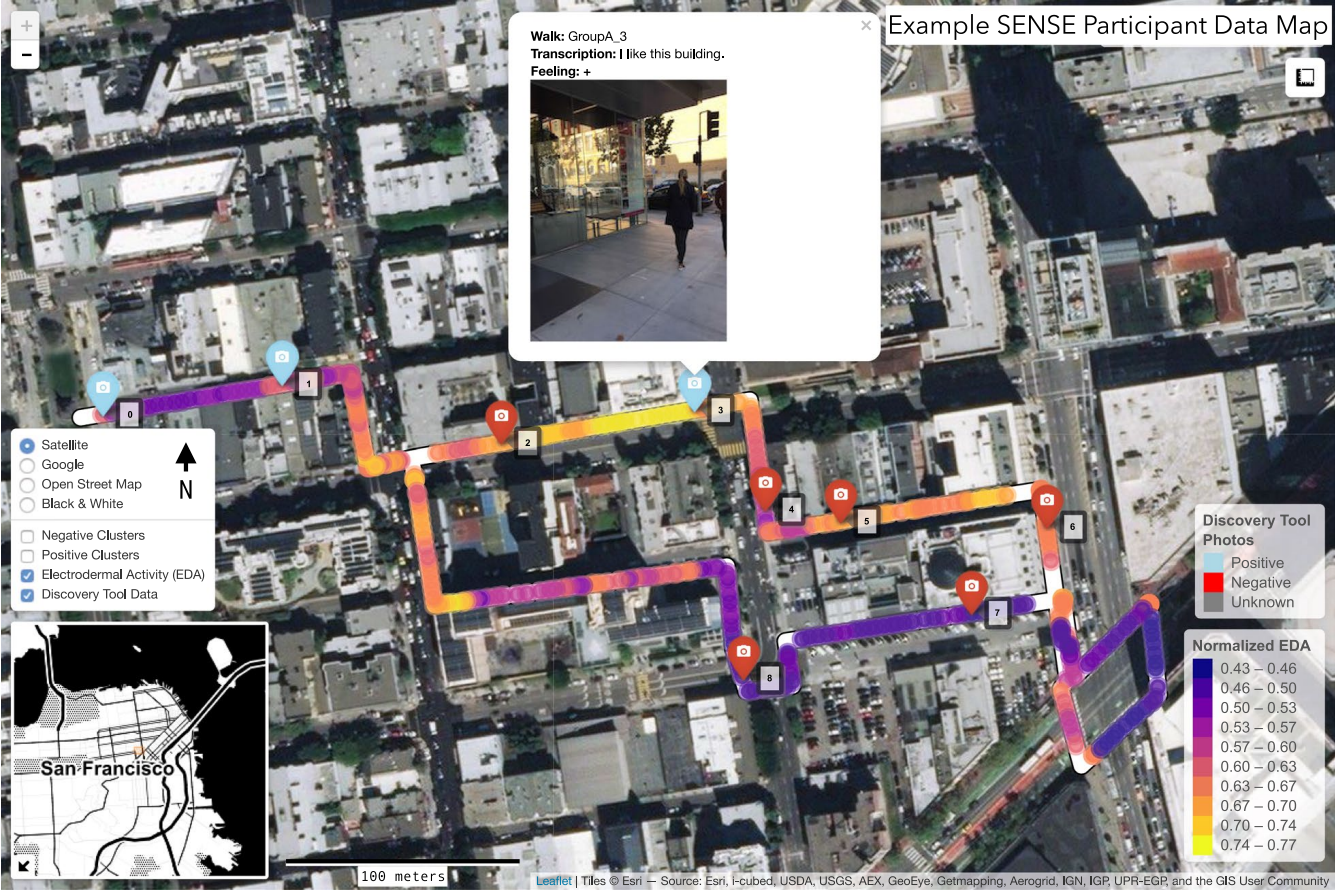
Example of an interactive webpage built for participants to view and interpret their data. The red and blue markers show where this specific participant took photographs with the DT app. The participant’s path is color-coded by their EDA level, from dark purple to yellow (low to high). The complete html file and underlying R code has been uploaded as Additional files 1 and 2

Visualizations of each individual participant’s walking route while using the DT app were generated with *leaflet,* an open-source JavaScript visualization library, which we deployed within the R software environment [45–47]. Markers indicating the location of photos/audio narratives taken were added to these maps, and two-dimensional binned kernel density estimates were calculated to visualize clusters of positive and negative photos (using the *bkde2D* function of the KernSmooth package) [48, 49]. The walking route was color-coded by participants’ relative EDA levels during their walk, with peaks illustrated as additional markers of sizes according to their amplitude. These web-based visualizations were shared with participants via email, and their feedback was solicited with an open-ended web-based survey. Interactive data maps were generated for participant feedback, but were not specifically analyzed as part of this study. These maps allowed participants to (1) reflect on data collected during the neighborhood walk; (2) see how EDA levels changed over the course of the walk in relation to objective neighborhood features (e.g., basemap and DT app photos); and 3) compare their data to other participants along the same route. An example data map is shown in Fig. 1, and an interactive example is provided as Additional file 1.

### Spatial and statistical analyses

The database underlying the participant walk map visualizations described above was imported to a geographic information system software, ArcMap 10.5.1 [50], where a variety of spatial data (listed below) from the City of San Francisco had been pre-assembled [51, 52]. Spatial joins were performed to assign each walking route GPS coordinate several fields from these local data, in addition to the biometric data used in the visualizations: distance to the nearest positive- and negative-rated DT photo, distance to the nearest street intersection (as possible points of interest in terms of high traffic/activity), parcel characteristics (e.g., current land use, age of buildings), and street characteristics (e.g., name, one/two-way traffic pattern, classification as a highway, major, secondary, or local street).

In addition to summary statistics of participants’ EDA data by different spatial characteristics, two additional analyses were performed. First, the significance of spatial clustering of positive and negative DT photos was assessed with the Optimized Hot Spot Analysis tool, which calculates the Getis-Ord Gi* local statistic (Gi*), a standardized measure of clustering for specified areal units (here, set as 5-m grid cells along the walk path) [53–55]. For this pilot, the top quintile of Gi* statistics were selected as the most highly clustered cells; this procedure was performed separately for positive and negative DT photos. A subsequent spatial join between these highly clustered cells and the participant walk data created a binary variable for observations inside or outside of a clustered cell.

Second, a linear mixed model was fit on geo-located biometric data observations using R (via *lmer* in the *lme4* package) to identify associations between the main outcome, participant EDA, with contextual walk measures as fixed effects [56, 57]: location inside or outside of a highly-clustered cell, location within 10 m of a street intersection, one/two-way traffic pattern and classification of street, land use of the nearest parcel, age of building on the nearest parcel, and the observation’s time during the walk. A random intercept was specified to account for grouping of the study design: biometric observations within individuals (14 participants) within groups (5 walk groups). To illustrate possible within-subject variations, simple linear regression models were fit for three participants for EDA outcomes and whether the observation was taken in positive or negative DT cluster.

## Results

**Fig. 2:**
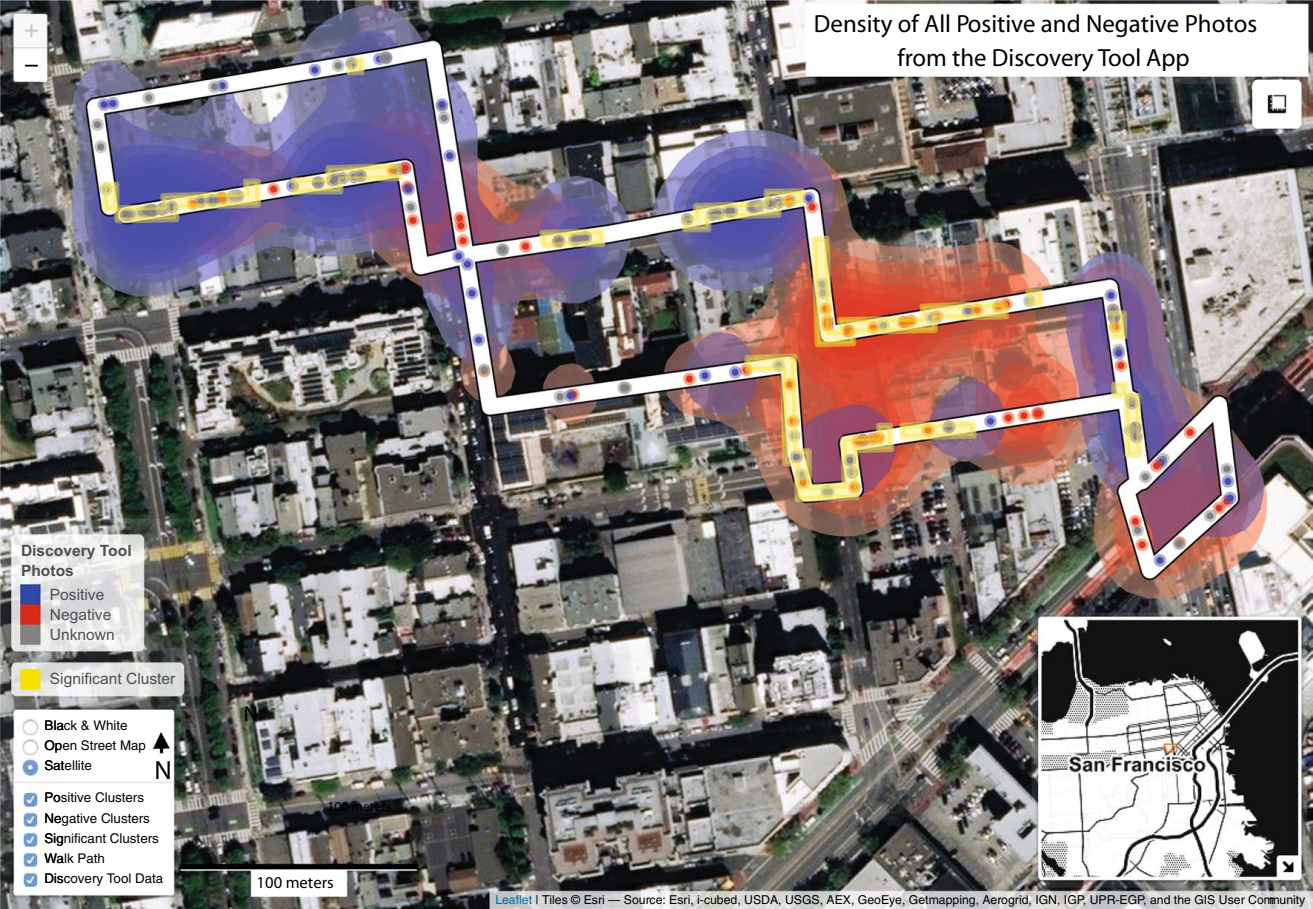
Heat map of positive and negatively-rated photographs taken with the DT app. Colors indicate if clusters of photographs were rated as positive (blue shading) and negative (red shading) by participants using the DT app. Darker shading indicates a higher density, and yellow cells indicate clustering of positive/negative photos per the Getis-Ord Gi* local statistic (top quintile)

In total, 14 adults (8 women and 6 men) who lived in the San Francisco Bay Area were recruited to participate as a convenience sample. Participants recorded 181 images (mean 15.1, SD 8.4) and 146 audio files (mean 12.2, SD 8.6) with the DT app, and 5416 geo-located biometric data observations were collected from 13 participants (approximately 416 observations per participant). Figure 2 illustrates the spatial distribution of photographs taken with the DT app by the positive/negative valence assigned by participants for the built environment features being captured.

**Fig. 3:**
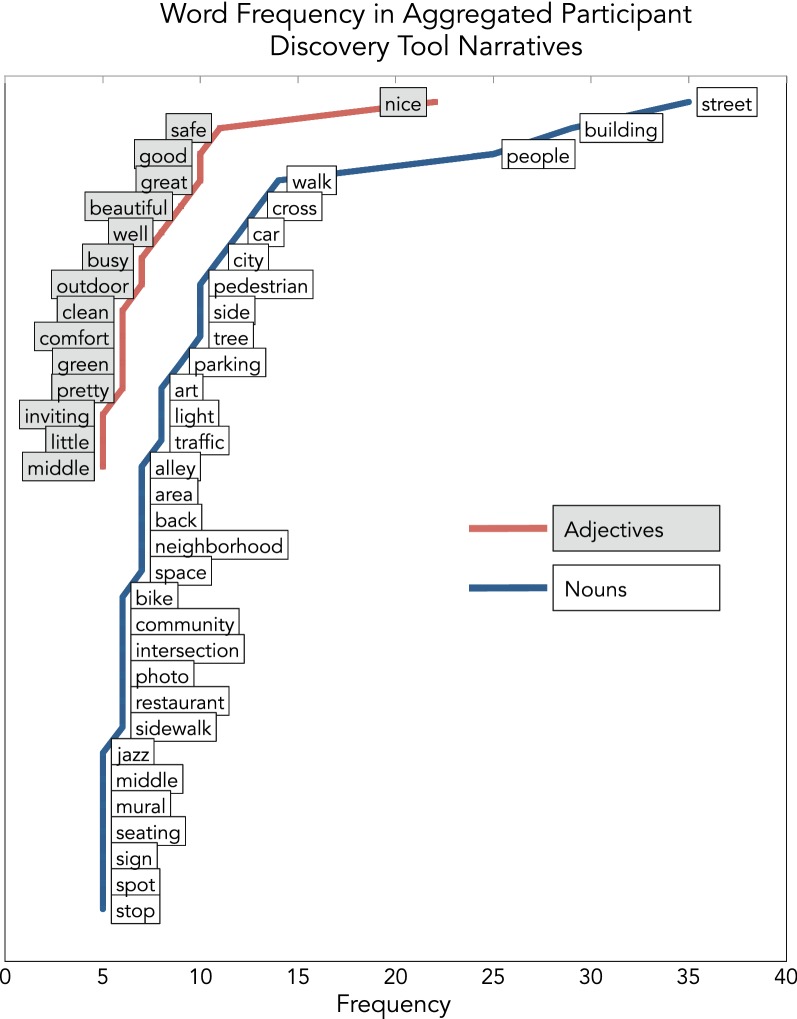
Visualization of nouns and adjectives with an overall frequency greater than five in all audio narratives

Among captured images that were tagged with a participant-coded valence (n = 131), just over half were positive (n = 77). Within-participant positive/negative valence ratios were similar, with most participants rating 53.0% of their images as positive (SD 21.4%). The average distance to a positive DT photo during a walk was 11.9 m, while the average distance to a negative photo was 14.7 m. In terms of the narratives participants used to explain their photographs in the DT app, several words were repeated frequently. The most common nouns used by participants in their audio narratives included “street” (n = 35), “building” (n = 29), and “people” (n = 25), while “nice” (n = 22), “safe” (n = 11), and “good” (n = 10) were the most common adjectives. Figure 3 provides a visualization of all nouns and adjectives used by participants with an overall frequency greater than five.

**Fig. 4:**
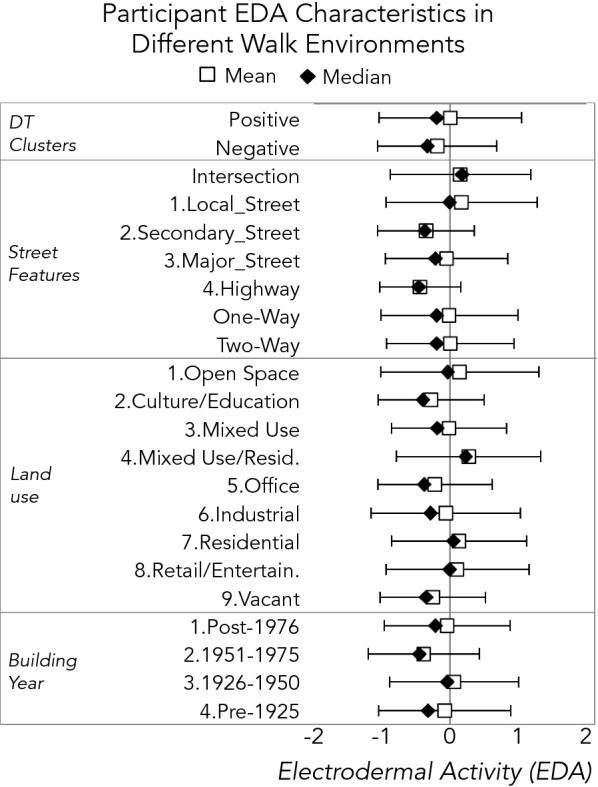
Average and median EDA level observed by different walk environments (SD shown in brackets)

Based on the Getis-Ord Gi* local statistic (estimated at a 95% confidence level), two significant clusters of positive DT photos comprised approximately 4.3% of the walk route (by distance), while negative photo clusters represented 2.7% of the route, also in two significant clusters. Both of the significant positive clusters occurred within the first half of the walk route, and both significant negative clusters occurred within the second half, though both negative and positive DT photos were taken on all except one street during the course of the walk. Table 1 summarizes the frequency of walk observations by a variety of environmental characteristics, including presence inside of a positive or negative cluster, and Fig. 4 displays summary statistics for electrodermal activity across these characteristics.

**Table 1:** Summary of walk observations by environmental characteristics

	n obs.	% Total obs.
*DT clusters*		
Inside positive	1122	21
Inside negative	1107	20
*Street features*		
Intersection	416	8
One-way	3608	67
Two-way	1804	33
Local	2182	40
Secondary	155	3
Major	2619	48
Highway	456	8
*Land use*		
Open space	315	6
Culture/education	357	7
Mixed use	312	6
Mixed use/residential	1241	23
Office	1716	32
Industrial	329	6
Residential	514	9
Retail/entertainment	248	5
Vacant	286	5
*Building age*		
Post-1976	1604	30
1951–1975	196	4
1926–1950	1261	23
Pre-1925	1737	32

Based on the linear mixed model, statistically significant positive associations were found between participant EDA and positive photo clusters (*B *= 0.14, *p *< 0.001), and significant negative associations were found between participant EDA and negative photo clusters (*B *= − 0.17, *p *< 0.001). This suggests that, on average, participants’ EDA was higher in areas where many participants documented more favorable features of the environment, and lower in areas where they documented less favorable features. Other significant associations between EDA and walk characteristics were also observed, including the street type (significantly lower for highway, major, and secondary streets compared to local streets, *p *< 0.001), and land uses compared to parcels designated as “open space” (significantly higher near mixed/residential [*p* < 0.001] and residential [*p* = 0.033]; significantly lower near office [*p* = 0.003] and vacant buildings [*p* < 0.001]). The age of buildings also was associated with EDA, with older buildings seeing higher observations, compared to the most recent buildings. All models, 95% confidence intervals, and coefficients are displayed in Table 2.

**Table 2:** Linear mixed model of participant EDA observations with group and participant-level random intercepts

	Electrodermal activity (EDA)		
	*B*	*CI*	*p*
*Fixed parts*			
Time on walk	0.20	0.17 to 0.24	*< .001*
Positive photo cluster	0.14	0.06 to 0.23	*< .001*
Negative photo cluster	− 0.17	− 0.25 to − 0.09	*< .001*
Intersection	0.05	− 0.04 to 0.15	.284
2-way street *(ref:1*-*way)*	0.15	0.07 to 0.23	*< .001*
*Street type (ref:Local)*			
Secondary	− 0.49	− 0.72 to − 0.27	*< .001*
Major	− 0.17	− 0.24 to − 0.11	*< .001*
Highway	− 0.47	− 0.61 to − 0.34	*< .001*
*Land use (ref: open space)*			
Cultural	− 0.11	− 0.31 to 0.08	.244
Mixed	0.10	− 0.11 to 0.30	.365
Mixed/residential	0.29	0.12 to 0.47	*< .001*
Office	− 0.24	− 0.40 to − 0.08	*.003*
Industrial	0.09	− 0.09 to 0.28	.329
Residential	0.18	0.01 to 0.35	*.033*
Retail/entertainment	0.01	− 0.18 to 0.20	.924
Vacant	− 0.38	− 0.55 to − 0.21	*< .001*
Missing	− 0.08	− 0.32 to 0.16	.498
*Building age (ref: 1976*–*present)*			
1951–1975	0.15	− 0.03 to 0.33	.107
1926–1950	0.10	0.01 to 0.18	*.030*
Pre-1925	0.09	0.02 to 0.17	*.011*
Unknown	0.32	0.16 to 0.48	*< .001*
(Intercept)	0.01	− 0.19 to 0.20	.932
Random parts			
σ^2^	0.854		
N_partid:(partgroup:time)_	14		
N_partgroup:time_	5		
N_time_	2		
Observations	5412		
R^2^/Ω_0_^2^	.119/.119		

Italic values are significant at *p* < 0.05

Exploratory linear regression models for three individual participants also showed significant relationships for presence in a positive or negative DT photo cluster, though they were of varying magnitudes and directions (see Table 3). Additionally, these regressions indicated that positive and negative clusters had far better explanatory value for some participants’ EDA than for others (e.g., R^2^ = 0.076 for Participant A3, vs. R^2^ = 0.263 for Participant B3).

**Table 3:** Linear models of participant EDA observations showing within-subject correlations with positive/negative DT clusters

	Participant A3			Participant B3			Participant E3		
	*B*	*CI*	*p*	*B*	*CI*	*p*	*B*	*CI*	*p*
(Intercept)	0.03	− 0.11 to 0.16	.680	− 0.23	− 0.35 to − 0.11	*< .001*	0.30	0.18 to 0.42	*< .001*
Positive cluster	0.35	0.10 to 0.60	*.007*	1.15	0.93 to 1.38	*< .001*	− 0.50	− 0.74 to − 0.26	*< .001*
Negative cluster	− 0.47	− 0.71 to − 0.22	*< .001*	− 0.17	− 0.40 to 0.06	.147	− 1.00	− 1.22 to − 0.77	*< .001*
Observations	362			347			397		
R^2^/adj. R^2^	.076/.071			.263/.259			.164/.159		

Italic values are significant at *p* < 0.05

## Discussion

In this first-generation pilot investigation, we successfully assembled diverse technologies to collect and visualize objective, perceived, and biometric data in an urban neighborhood context. These data collection methods provide researchers with a means of investigating both group and individual-level responses to different environmental conditions.

In the case of the urban neighborhood walked by our participants, common perceptions of the built environment were observed, both in terms of the repeated terms captured in DT narratives and the significant clustering of positive and negative DT photos. At the group level, linear mixed model testing confirmed that the average participant EDA levels observed inside a positive cluster were significantly higher than those observed elsewhere on the walk. Conversely, EDA observations inside negative DT photo clusters were significantly lower than those from outside them. Further exploration is needed to understand the dimensions of this relationship, though these preliminary statistical associations suggest that participants’ ratings of the built environment were reflected in their stress responses. Additionally, significant correlations between objective measures of the built environment, such as land use and street type, and EDA also highlight potentially influential relationships that could be more carefully tested with additional participant walks.

**Fig. 5:**
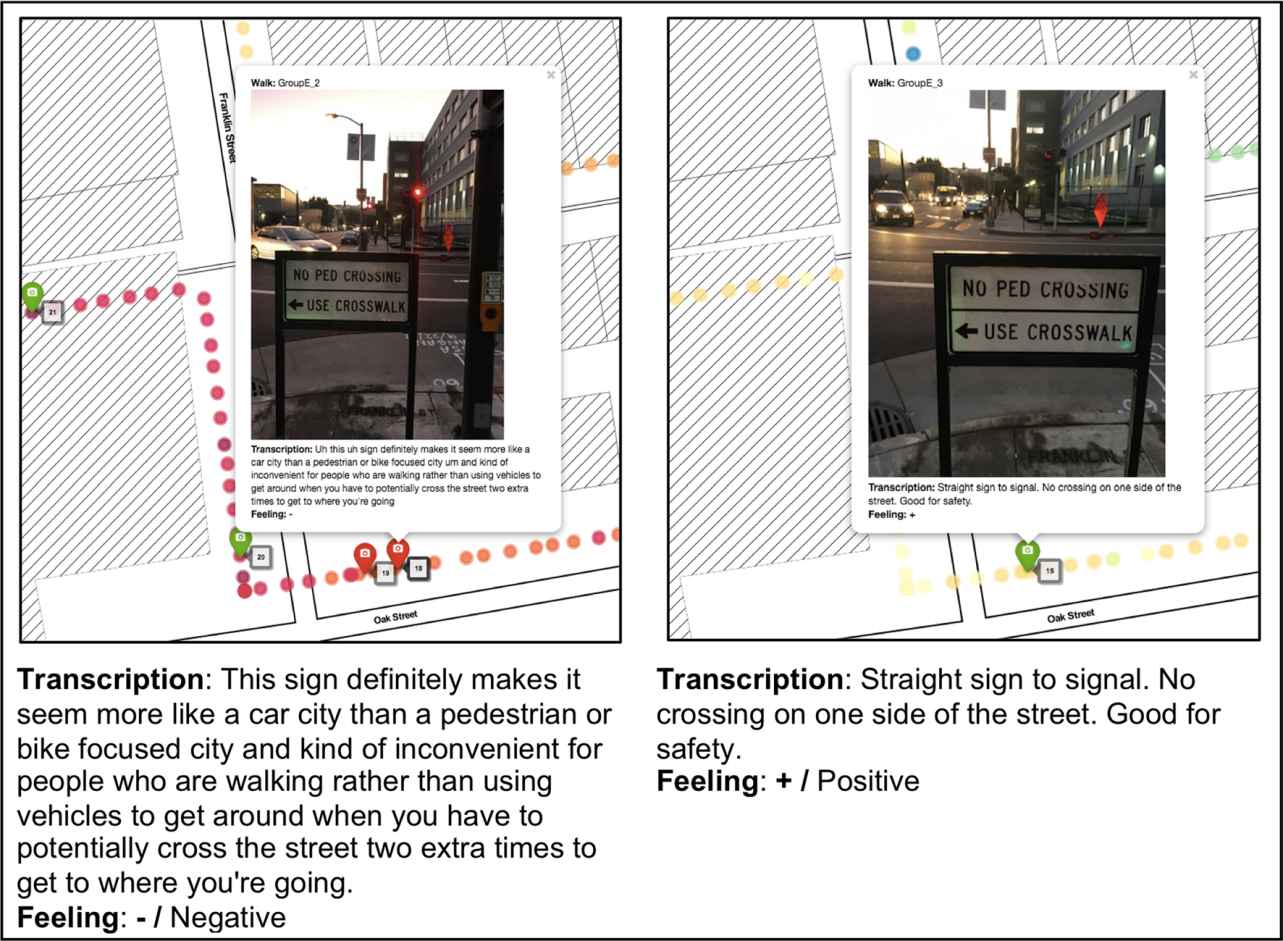
Participant data maps. While these two participants also documented the same feature, they gave it different ratings and descriptions in terms of it being a positive or negative aspect of the built environment

These data also illustrated the value of multi-dimensional measurement at individual *and* group levels. While at least some participants may have been motivated to document similar built environment features, this did not mean that their interpretations were identical. For example, as shown in Fig. 5, two participants on the same walk captured images of a particular intersection that did not allow pedestrians to cross on all sides. For one participant, this represented a barrier, while the other rated it as a positive feature. While this was not a common occurrence, it is possible that future studies with additional participants, better measures of participant demographics (e.g., age, gender, length of residence in/around the city, etc.) or a focus on a narrower geographic area will find similar discrepancies between individual assessments of the same feature. Furthermore, as Table 3 illustrated, the strength of the relationship between EDA and participant-rated DT photos may not be consistent across all participants. Ultimately, this example underscores the importance of combining both objective and perceived built environment information in assessments.

### Limitations

Another study limitation was the small number of residents included in this initial feasibility study to develop a systematic process for collecting and analyzing diverse types of data geo-located data. Though the participant sample is small, these individuals produced a relatively large database of qualitative data in terms of photo (n = 181) and audio narratives (n = 146). Similar studies using the Discovery Tool application have found even small groups of participants (e.g., 8–10) are able to reach thematic saturation when identifying positive and negative features of a particular built environment [32]. Additionally, the fine-grained biometric data collection adds thousands of additional data points in which these photos and audio narratives can be contextualized. The methods we have described are easily scalable to accommodate many more participants, should future researchers desire to integrate them into crowd-sourcing initiatives, as in other quantified self projects [27].

In terms of logistical challenges, we encountered issues with mobile app stability during some walks because of the amount of image/audio data being collected, which resulted in the loss of photo and audio data for some participants. Our research team was able to retrieve or recreate some photos with Google Street View, and participant experiences informed subsequent developer updates to the DT app (e.g., enabling reliable capture of larger quantities of images and audio narratives) along with an accompanying troubleshooting guide for new users.

Several methodological questions were also raised during the course of the pilot, and should be considered in future research. First, the potential effects of having participants walk in groups, as undertaken in this study, versus independently, are worth further consideration. It is likely—and was observed by some participants—that individuals felt more inclined to take photographs when they observed others in their group doing so. One participant described this circumstance in an audio narrative about a small church building along the route: “I find that when the person I’m on a walk with takes a photo of something, I want to take a photo of the same thing. But it’s true, this blue building is pretty excellent” (see Additional file 3).

Another methodological question relates to the use of the Empatica E4 sensor. While we used a 10-min pre-walk period to obtain a reasonable baseline measurement, it is possible that longer time periods or data collected under different circumstances (e.g., walking vs. standing or sitting) may yield more complete or interpretable measures for participants outside of laboratory environments. Other researchers using the Empatica sensor may find additional utility in the multi-dimensional data it creates to identify “signals” within the EDA data, versus “noise” possibly caused by a participant’s motions, perspiration due to exertion, or other factors. The availability of such resources as the “EDA Explorer,” which integrates the sensor’s 3-axis accelerometry, skin temperature, and EDA data to more precisely estimate EDA changes, suggest that device developers are considering the implications of collecting electrodermal data in ambulatory settings, perhaps a sign of future guidance on this topic [58, 59].

The neighborhood walk route was also the subject of methodological deliberations. While having participants walk the same route allowed for a more direct comparison between participants’ DT app and biometric data, it also imposed a relatively arbitrary constraint on what has often been a more free-form built environment assessment in other *Our Voice* projects [32]. Additionally, this pilot study allowed participants to investigate a variety of urban spaces, though future iterations may pursue a more in-depth exploration of a single, specific space, such as a park or plaza. Quantified self researchers may also find utility in collecting individual geo-tagged biometric stress data over several hours or days in future “n-of-1” studies or interventions [60]. Importantly, the method described here is sufficiently flexible to be tailored to the research questions of new projects, but provides key capabilities for collecting and visualizing different kinds of objective and perceived participant data.

Ultimately, the questions raised during the study may also prompt deeper qualitative analyses. As a participant eloquently summarized in one of her audio narratives:

There’s this interesting dichotomy that I don’t know how to express in a photograph which is the pleasure of being in a complex urban environment balanced with a serenity and beauty. Both are pleasing, one is more intense which maybe might make you… maybe my biometrics feel aggravated or disoriented in some ways but that is one of the reasons we love cities so we should not optimize out a sense of complexity and chaos because that too is beautiful.

These pilot data provide a starting point for researchers and citizen scientists to “triangulate” between the objective, perceived, and embodied experiences of built environment spaces in ways that could lead to new insights, including the beauty of “complexity and chaos.”

## Conclusion

Identifying elements of one’s environment—both observable and unobservable—that contribute to allostatic load presents new opportunities to engage community residents in collecting and analyzing their personal data to mobilize potential environmental improvements. The current investigation provides a systematic process of collecting these three types of data, and lays a foundation for future spatial and statistical analyses in addition to more in-depth interpretation of how these responses vary within and between participants. This type of multi-dimensional data collection procedure could be integrated into future built environment or quantified self research projects where biometric data are also of interest to community members, and our open-source mapping technology (R code provided as Additional file 2) allows for easier replication in different settings and projects. It sets the stage for additional research aimed at better understanding—both qualitatively and quantitatively—how objective and perceived elements of the built environment influence our “lived” experience in different settings, which may impact people’s stress as well as well-being and quality of life.

## Additional files

**Additional file 1.** Interactive Data Map. Geospatial visualization of participant data as an html file, suitable for viewing in a web browser.

**Additional file 2.** R code. Sample R code for processing and visualizing participant Discovery Tool and biometric data.

**Additional file 3.** Example participant data maps. These two participants were on the same walk and took photographs of the same building. One (at right) observed that they noticed this influence: “I find that when the person I’m on a walk with takes a photo of something, I want to take a photo of the same thing. But it’s true, this blue building is pretty excellent.”

## Abbreviations

AL: allostatic load
DT: Discovery Tool
EDA: electrodermal activity
EEG: electroencephalogram
SD: standard deviation
