# Supplementary material for: Stress experiences in neighborhood and social environments (SENSE): a pilot study to integrate the quantified self with citizen science to improve the built environment and health
Source: Int J Health Geogr. 2018 Jun 5;17:17. doi: 10.1186/s12942-018-0140-1 (PMC5989430; doi:10.1186/s12942-018-0140-1)
Supplement: Supplementary file 1 — Additional file 1. Interactive Data Map. Geospatial visualization of participant data as an html file, suitable for viewing in a web browser. [file 12942_2018_140_MOESM1_ESM.html]

leaflet


 
