# Supplementary material for: Stress experiences in neighborhood and social environments (SENSE): a pilot study to integrate the quantified self with citizen science to improve the built environment and health
Source: Int J Health Geogr. 2018 Jun 5;17:17. doi: 10.1186/s12942-018-0140-1 (PMC5989430; doi:10.1186/s12942-018-0140-1)
Supplement: Supplementary file 3 — Additional file 3. Example participant data maps. These two participants were on the same walk and took photographs of the same building. One (at right) observed that they noticed this influence: “I find that when the person I’m on a walk with takes a photo of something, I want to take a photo of the same thing. But it’s true, this blue building is pretty excellent.” [file 12942_2018_140_MOESM3_ESM.pdf]

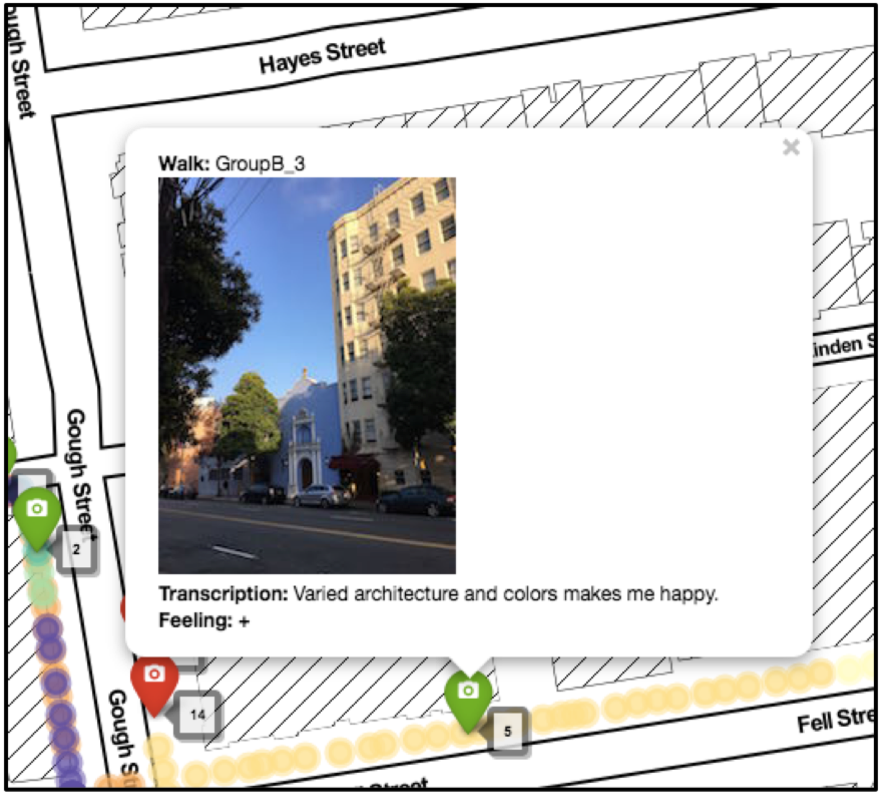

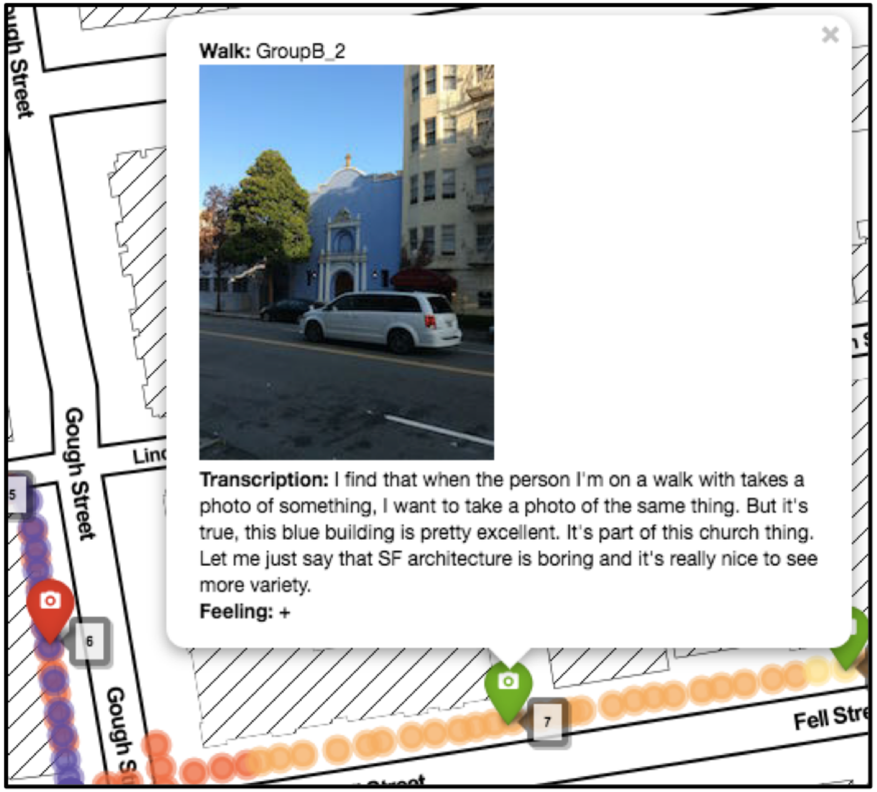

Transcription: Varied architecture and colors make me happy.
Feeling: +

Transcription: I find that when the person I'm on a walk with takes a photo of something, I want to take a photo of the same thing. But it's true, this blue building is pretty excellent. It's part of this church thing. Let me just say that SF architecture is boring and it's really nice to see more
variety.
Feeling: +
